# Central Venous Access and the Risk for Thromboembolic Events in Patients Undergoing Neoadjuvant Chemotherapy and Radical Cystectomy for Muscle-Invasive Bladder Cancer

**DOI:** 10.3390/life12081198

**Published:** 2022-08-06

**Authors:** Harriet Rydell, Ylva Huge, Victoria Eriksson, Markus Johansson, Farhood Alamdari, Johan Svensson, Firas Aljabery, Amir Sherif

**Affiliations:** 1 Department of Surgical and Perioperative Sciences, Urology and Andrology, Umeå University, 90187 Umeå, Sweden; 2 Department of Clinical and Experimental Medicine, Division of Urology, Linköping University, 58183 Linköping, Sweden; 3 Department of Urology, Västmanland Hospital, 72189 Västerås, Sweden; 4 Department of Statistics, Umeå School of Business, Economics and Statistics (USBE), Umeå University, 90187 Umeå, Sweden

**Keywords:** complications, cystectomy, central venous catheters, neoadjuvant therapy, thromboembolism, urinary bladder neoplasms

## Abstract

Thromboembolic events (TEE) are high-risk complications in patients undergoing neoadjuvant chemotherapy (NAC) and radical cystectomy (RC) for urothelial muscle-invasive bladder cancer (MIBC). The purpose of the study was to investigate any differences in TEE-incidence, comparing peripherally inserted central catheter (PICC) versus a totally implanted port (PORT) as CVA (central venous access) during NAC. We identified 947 cystectomized MIBC-patients from four Swedish medical centers in 2009–2021. Inclusion criteria were cT2-T4aN0M0 and 375 patients were finally eligible and evaluated, divided into: NAC-administered (*n* = 283) resp. NAC-naïve-NAC-eligible (*n* = 92), the latter as tentative control group. Data on TEEs and types of CVA were retrospectively collected and individually validated, from final transurethral resection of the bladder tumor (TUR-B) to 30 days post-RC. Adjusted logistic regression and log rank test were used for statistical analyses. Amongst NAC-administered, 83% (*n* = 235) received PICCs and 15% (*n* = 42) PORTs. Preoperative TEEs occurred in 38 PICC-patients (16.2%) and in one PORT-patient (2.4%), with 47 individual events registered. We found a significantly increased odds ratio of TEE in NAC-administered PICC-patients compared to in PORT-patients (OR: 8.140, *p*-value: 0.042, 95% CI 1.078–61.455). Our findings indicate a greater risk for pre-RC TEEs with PICCs than with PORTs, suggesting favoring the usage of PORTs for MIBC-NAC-patients.

## 1. Introduction

Bladder cancer is the ninth most common cancer worldwide and ranks 13th in terms of deaths, with smoking as its major risk factor [1,2]. Approximately 25% of all patients present with muscle-invasive cancer (cT2) at diagnosis. The treatment alternatives today depend on, tumour staging, clinical staging, patient age and comorbidity. The gold standard treatment for muscle-invasive bladder cancer (MIBC) is radical cystectomy (RC) preceded by neoadjuvant chemotherapy (NAC) in medically fit and eligible patients [3].

The prognosis of urothelial MIBC is relatively poor with a five-year survival around 50%, for all clinical stages T2–T4 following RC [3]. Cisplatin-based neoadjuvant combination (NAC) significantly increases survival for a subgroup of these patients. For the chemo-sensitive sub-group, there is an absolute risk reduction (ARR) of 31% for death in completely downstaged patients (pT0N0M0) at five years median observation time, compared to cystectomy-only patients with complete downstaging. Thus, complete response (CR) serves as a surrogate marker for improved survival [4].

In the last 10 years, since the national introduction of NAC in Sweden, an increasing fraction of hospitals in Sweden have started to implement NAC for urothelial MIBC-patients. The most common alternatives for chemotherapy are either a combination of methotrexate, vinblastine, doxorubicin, and cisplatin (MVAC) given in 3–4 cycles, or gemcitabine and cisplatin/carboplatin (GC) for a total of 3–4 cycles [5]. Inclusion criteria for NAC, according to the Swedish guidelines, are biological age ≤ 75 years, intact renal function with eGFR > 55–60, no significant hearing impediment and acceptable comorbidity [6].

For non-responders to NAC, there will be a cystectomy-delay, in which the patients risk receiving largely ineffective treatment for 2.5–3 months prior to radical surgery. Retrospective studies evaluating the outcomes of NAC in regard of residual bladder cancer have reconfirmed that patients with completely downstaged tumours (CR), following transurethral resection of the bladder tumor (TUR-B), NAC, and RC, have significantly improved survival projections compared to NAC-non-responders [7,8,9]. These findings confirm the results of the post hoc analysis of the Nordic combined randomized prospective trials [4].

Since NAC has a level 1 recommendation and is regularly submitted to all eligible MIBC-patients, it is of great importance to optimize and ensure a good treatment-quality and to minimize the risk of adverse events (AEs). Although a successful and relatively well-tolerated treatment, the AE-frequency during NAC is considerable, shown recently by Eriksson et al. who showed that approximately 96% of NAC-administered patients suffered some form of AE during therapy. Amongst AE-types, a thromboembolic event (TEE) is considered being one of higher severity [6,10,11]. MIBC-patients have an established high risk of TEEs, with previous findings demonstrating an increased probability both treated and untreated with NAC [12]. Cisplatin-based NAC provides an even greater TEE-risk, with the theory of induced endothelial damage, thus placing MIBC-patients in a considerably higher risk-group [13]. However, the malignancy itself is also a contributing factor for TEE which further adds to the increased risk [14].

It is of great importance to have a reliable and safe long-term central venous access (CVA) in place, when undergoing chemotherapy. Peripherally inserted central catheters (PICC) and totally implanted chest ports (PORT) are both routinely used for this purpose in many centres. In Sweden, which of the two CVA types a patient receives is based on which clinical centre initiates NAC. The same patient could potentially receive either type, provided no contraindication, but is assigned with either PICC or PORT, depending on local routines. In Sweden, there has been an increase in the use of PICCs over the past decades due to the considered lower cost, insertion availability, perceived patient safety, and ease of removal when compared to other implanted CVA-devices, such as tunnelled catheters and PORTs [15,16]. The insertion and removal of PORTs must be performed under sterile conditions; by a physician in an operating theatre (OT) or at an interventional radiology (IR) facility. PICCs, on the other hand, are often inserted by specialist nurses in the outpatient clinic. The increased use of PICCs in cancer patients has occurred despite the paucity of controlled trials. Thus, several vascular access guidelines are unable to recommend one device over the other, due to insufficient evidence [17,18,19,20,21]. The PICCPORT trial randomised 399 patients with non-haematological malignancies and showed that there were 8% incidence rate of catheter-related deep vein thrombosis (CR-DVT) in the PICC-group and 1% in the PORT-group (difference = 7%; 95% confidence interval 2–12%). The PICCs were associated with more adverse events (DVT, line occlusion, infections, and mechanical events) as a whole, when compared to PORTs [5].

Our hypothesis was that when MIBC-patients planned for cystectomy undergo NAC, the rate of adverse thromboembolic events was higher if patients had a PICC compared to those having a PORT. Thus, the primary endpoint of our study was to investigate any differences in TEE-incidence between the PICC-and PORT-groups, and to determine the time to TEE in both groups. In addition, a secondary objective was to evaluate TEE-incidence in NAC-eligible patients, with neither type of CVA.

## 2. Materials and Methods

### 2.1. Patient Population

Radically cystectomized patients, derived from an extensive database spanning the years 2009–2021, were retrospectively analyzed in this multicenter study with treatment provided from one of the four participating centers around Sweden; Norrlands Universitetsjukhus in Umeå, Länssjukhuset in Sundsvall, Västmanlands sjukhus in Västerås and Universitetssjukhuset Linköping. After inclusion criteria, urothelial MIBC, and clinical staging cT2-T4aN0M0, 710 patients out of the 947 screened were evaluated. Both urothelial as well as urothelial mixed-type tumors where included, with 17 patients excluded because of ineligible histopathology. As the study’s main objective was the analysis of NAC-administered patients, those who did not receive this treatment nor fulfilled criteria for NAC-eligibility were excluded and termed as non-NAC-patients (*n* = 335), resulting in a final study population of 375 patients whom, depending on therapy, were divided into the study’s two main subgroups: the NAC-administered group (*n* = 283) and the tentative control group, the NAC-eligible group (*n* = 92).

### 2.2. NAC-Eligibility

**Figure 1:**
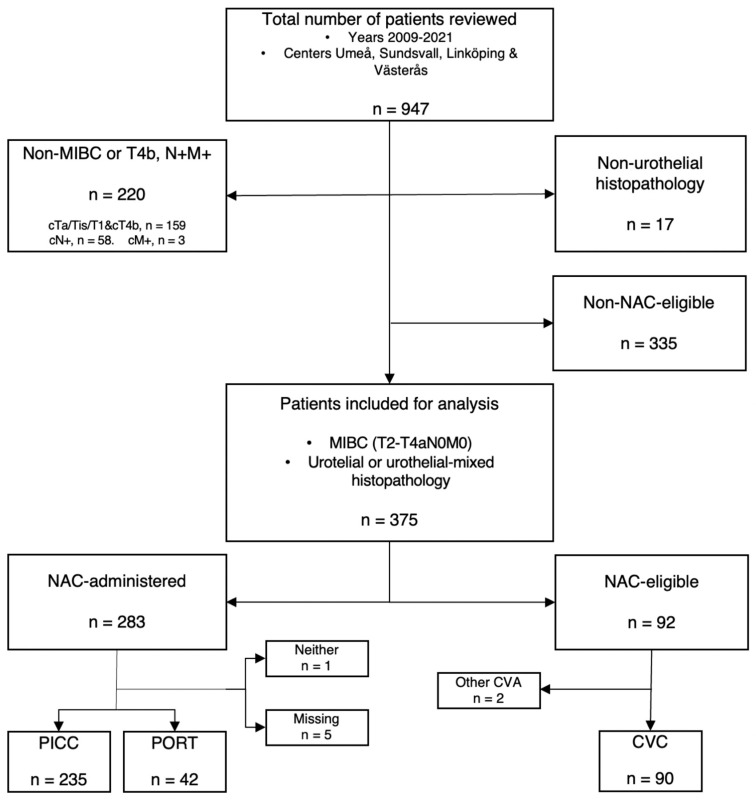
Flowchart of study population. CVA; central venous access. PICC; peripherally inserted central catheters. PORT; centrally inserted totally implanted vascular access port-a-cath. MIBC; muscle invasive bladder cancer. CVC; central venous catheter.

NAC was not common practice MIBC throughout all of Sweden until recent years and several and patients who at the time were eligible candidates never became recipients but would have received NAC by today’s guidelines. For example, in the Umeå cohort, 17% were NAC-treated in 2009 compared to 71% in 2021. NAC-eligible patients are considered being a fitting control group as TEE-incidences amongst them can be established *versus* incidences in NAC-administered, with as few confounding factors as possible. To establish which patients would have been offered NAC during the earlier years, each patient was individually evaluated, with the guidance of senior urologists and by pre-determined NAC-eligibility-criteria. Patients had to be <75 years old, with an eGFR > 50 or creatinine < 100 and Charlson Age Comorbidity Index (CACI) of 6 or less, to qualify as NAC-eligible. However, any clinical decision taken by the responsible clinician or by the multidisciplinary team (MDT) was considered as being the definite verdict regarding each patients’ eligibility. The selection of the study population is shown in Figure 1. CACI, which was individually calculated, includes variables, such as age, history of cardiopulmonary disease, diabetes, and cancer. CACI provides a standardized 10-year survival prediction and allows for an easy comparison between individuals.

### 2.3. Data Collection & TEE-Definitions

Data from individual medical journals was extracted and added to an already considerably large database, covering multiple clinicopathological variables; both general patient-characteristics such as age, gender, smoking habits, BMI, ASA, CACI, in addition to specifics on antithrombotic medication, NAC-details, type and insertion-date of CVAs, and TEE-incidences. The observation time was final TUR-B to 30 days post-RC; enabling comparison between the two subgroups of NAC-administered patients and the patients of the tentative control group, since the latter had neither PORT nor PICC and thus lacked an insertion date of either.

Information on type of NAC-regime, number of cycles, dates on administrations, adverse events and clinical responses were extracted. Due to some patients receiving NAC at their local hospital, dates on NAC-administration or CVA-insertion could be unavailable and therefor left as missing data. For PICC-patients who had a missing date for PICC-insertion, but an established date in the medical records for NAC-initiation, the latter was used as an insertion date in accordance with clinical routine in which PICC placement most often occurs on the same day as, or the day before, the start of NAC. One patient, because of a TEE anatomically related to the PICC, had the PICC replaced with a PORT, but is in the study classified as a PICC-patient-only.

TEE-incidences were individually dated and registered by type and severity. Events could be diagnosed either by clinical status alone, or alongside radiological visualization. Both symptomatic events and TEEs diagnosed incidentally were included. Pulmonary embolism (PE), DVT, TEEs anatomically related to the CVA, angina/myocardial infarction (MI), transient ischemic attack (TIA)/stroke, and thromboembolism were the included TEE-types, with one TEE classified as “other” being a kidney infarction occurring in a PICC-patient post-RC. A TEE was deemed anatomically related to the CVA only if stated as such by the attending radiologist. A TEE-incidence in one patient was registered as a single case which statistical analyses are based upon, in addition to each singular event being cumulatively calculated. If one patient suffered multiple events, the first event was used in analysis, with the exception in two patients, where the first patient’s initial TEE developed before CVA-insertion and the second patient’s between insertion and cystectomy; one PICC- and one PORT-patient, respectively. For them, the second TEE was analyzed instead. No patients developed a TEE before CVA-placement as their only event. Common Terminology Criteria for Adverse Events (CTCAE) measures the severity of adverse events, with a general scoring of 1–2 indicating asymptomatic/mild disease, grade 3 meaning an event of further severity with the need of intervention, grade 4 signifying intensive care was needed, and grade 5 death related to event. However, the specific CTCAE-scoring available for each different type of thromboembolic adverse event was used. No scoring system for the TEE-risk was used, such as the Khorana risk score, with recent findings indicating that a high Khorana score cannot predict TEE-risk in MIBC-patients receiving NAC [22]. Instead, our strict inclusion criteria allowed for a relatively cohesive patient population.

Antiplatelet, anticoagulants, and temporary antithrombotic prophylaxis were registered as separate variables. The clinical practice is to discontinue antithrombotic drugs peri-operatively and was therefore still considered as a current medication in the study. If any antithrombotic drugs were discontinued during the NAC-period after TUR-B, the patient was not registered as being on any antithrombotic medication. Routines for TEE-prophylaxis were the same for all included centers regardless of CVA-type. LMWH was provided a limited time post-operatively, and no prophylaxis was offered pre-operatively.

#### 2.3.1. Statistics

Statistical analyses were conducted by IBM SPSS v. 28 and all tests were two-sided with a significance level of 5%. For different time-periods, we have used adjusted and unadjusted logistic regression, for evaluating how TEE-incidences depends on type of CVA. The tests were adjusted for CACI, if the patient was a current smoker at the time of TUR-B, and for type of antithrombotic prophylaxis, if any. CACI was modeled as interval-variable. We visualized time to TEE for PICC- and PORT-groups with Kaplan–Meier curves and made inferences regarding the difference between the curves with the log rank test. Additionally, logistic regression was also used for analysis of the relationship between thrombosis prophylaxis and TEE. Timepoints for CVA-placement for some patients were missing. These lost data are assumed missing completely at random due to registration errors.

#### 2.3.2. Ethics

Approval was received from the Ethics Review Board (EPN-Umeå): DNR: 2013-463/3 (with the latest specific amendment for this study from EPM: DNR 2019-04291). Data-collection was strictly carried out according to GDPR-regulations.

## 3. Results

Out of 947 patients eligible for analyses, 375 were evaluated and incorporated in the final evaluation. The mean age of the cohort was 67 years (40–80), mean BMI was 26, and 77% were males. Furthermore, 283 were NAC-administered patients, who received chemotherapy for MIBC, whilst 92 patients were NAC-eligible NAC-non-receivers and set as the tentative control group. If NAC was given, each patient received one optional type of CVA; either a PICC (*n* = 235) or a PORT (*n* = 42). However, one patient only received a peripheral vein catheter (PVC) for NAC-administration. In addition, some patients received a CVC perioperatively despite their initial PICC or PORT, depending on the attending clinicians’ choice. In the control group, all patients received a CVC perioperatively (*n* = 90), except one patient who was supplied with a PICC and one patient with a PVC only. Due to some medical journals lacking a date of CVA-insertion, 7% (21/283) of CVA-dates are counted as missing data. Patients were either cystectomized or received their NAC-treatment at one of four Swedish medical centers; 162 in Umeå, 49 in Sundsvall, 47 in Västerås, and 109 in Linköping. The most utilized NAC in both PICC- and PORT-groups was MVAC (*n* = 192, 82% and *n* = 26, 62%), followed by Carboplatin-Gemcitabine (*n* = 15, 6% and *n* = 7, 17%) and MVEC (*n* = 14, 6% and *n* = 0, 0%). Additionally, 31 patients did not receive any of the mentioned regimes and are thus classified as “other” regimen (*n* = 17, 7% and *n* = 11, 26%). Four patients experienced side-effects from their initial regimen leading to a regimen-change and are therefore represented in two NAC-groups. Mean number of NAC-cycles were 2.9 in the PICC-group and 3.0 in the PORT-group, respectively. Specific patient characteristics are further presented in Table 1.

During the time period of TUR-B to 30 days post-surgery, 83 individual TEEs occurred, of which 71 events (85.5%) were within the NAC-group; 66.2% (*n* = 47) pre-RC. Further, 38 PICC-patients (16.2%) and ONE PORT-patient (2.4%) had TEEs during the time frame from TUR-B to RC, with 51 (61.4%) individual TEEs registered, out of which five PICC-patients had two TEEs each and one PICC-patient had a total of three TEEs. The most common type of TEE in the PICC-group was PE (*n* = 21). Mean time from CVA-insertion to TEE in the PICC group was 35.5 (6–95) days. DVTs and thrombophlebitis developed in three and in seven PICC-patients respectively, and one patient suffered from angina/MI. Further, 13 patients had a TEE anatomically related to the PICC. For example, one PICC-patient, a 74-year-old man, presented with a T3N0M0G3 tumor and received two NAC-cycles after which he developed a thrombosis related to the PICC, with a PE as a subsequent finding. NAC was terminated and seven positive lymph nodes were found postoperatively. That patient passed away two years after RC.

The single PORT-patient who developed TEEs was a 77-year-old man without a previous medical history or any medication. He experienced a total of two TEEs, firstly a PE after TUR-B but before CVA-insertion, and secondly a CT-verified TEE anatomically connected to the PORT, during NAC. The second TEE occurred 35 days after PORT-insertion whilst the patient had ongoing heparin treatment, which was introduced after the initial PE, thus prior to PORT-insertion. In the tentative control group, three patients (3.3%) developed a total of four TEEs, with one patient suffering both a DVT and a PE. The most common CTCAE grades were 2–4 and no patient died during the NAC-period. Details on all TEEs from TUR-B to RC are presented in Table 2.

**Figure 2:**
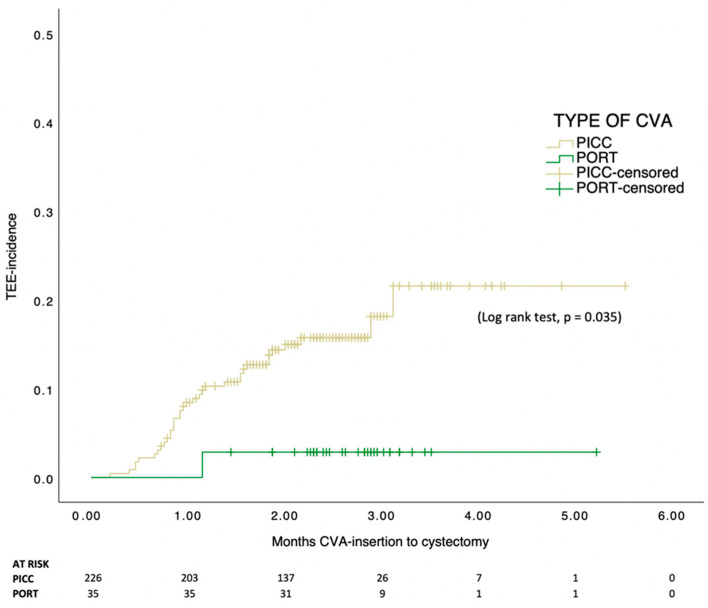
Time to first TEE-incidence in PICC- and PORT-patients displayed over time. Patients at risk stated in (n). CVA; central venous access. PICC; peripherally inserted central catheters. PORT; centrally inserted totally implanted vascular access port-a-cath. TEE; thromboembolic event.

PICC-patients, during NAC prior to RC, had a significantly increased risk of TEE incidence with an odds ratio of 8, compared to patients with a PORT (*p*-value: 0.042, CI 95%: 1.078–61.455). A significant difference was seen between these CVA-groups where PICC-patients had a higher TEE-incidence over time, compared to PORT-patients (log rank test, *p*-value: 0.035) (Figure 2).

**Figure 3:**
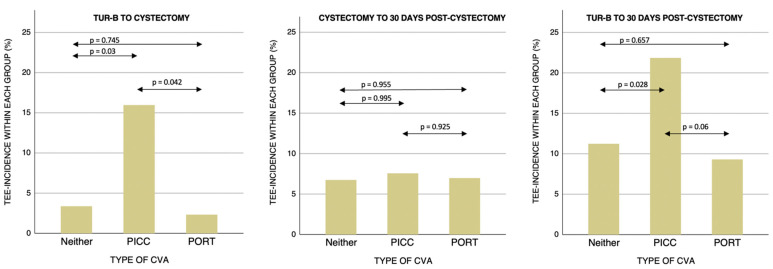
TEE-incidences during stratified time-periods, presented as % of patients with TEE within each CVA-group. P-value for the difference of TEE-risk between each group using logistic regression, adjusted for CACI, thrombosis prophylaxis and current smoker. CVA; central venous access. PICC; peripherally inserted central catheters. PORT; centrally inserted totally implanted vascular access port-a-cath.

**Figure 4:**
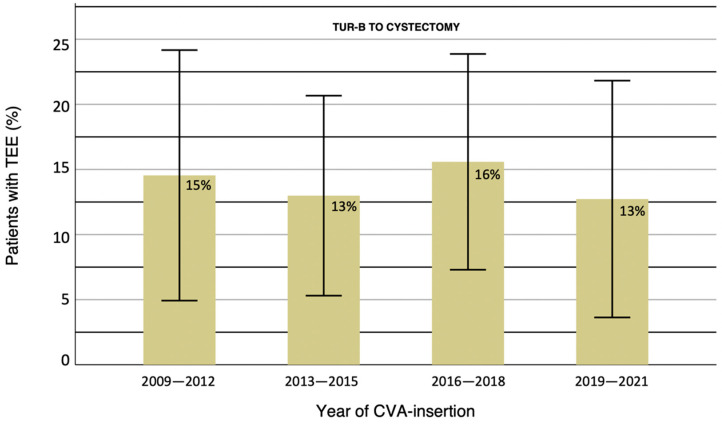
TEE-incidences in all CVA-groups during the complete observation-time. Data is represented as mean % of each stratification of years. CI 95% for mean-value. TUR-B; transurethral resection of bladder. TEE; thromboembolic event. CVA; central venous access.

Adjusted analysis showed no individual variable with significance to TEE incidence. No statistical significance could be found during any other period; RC to 30 days postoperatively (*p*-value: 0.925), nor TURB to 30 days postoperatively (*p*-value: 0.06) (Table 3 & Figure 3). Crude logistic regression tests for prophylaxis showed no association to TEE incidence during any period for either an antiplatelet-drug (*p*-value: 0.186) or an anticoagulant-drug (*p*-value: 0.694). No relation could be found between overall TEE-incidence during the time from TUR-B to RC or specific year of CVA-insertion (*p* = 0.958), with a mean TEE-incidence of 14% each year between 2009 and 2021 (Figure 4).

Furthermore, no difference was observed between TEE-incidence or grade of TEE-severity during the time between RC up to 30 days post-surgery in any of the CVA-groups. In addition, 17 PICC-patients (7.2%) had postoperative TEEs, with two patients passing away due to the TEE. Three PORT-patients (7.1%) had TEEs postoperatively, of which one died due to MI. In the tentative control group, six patients (6.6%) had TEEs post-RC, of which one patient, who had a DVT as well as other cardiopulmonary complications, died.

## 4. Discussion

Our findings present a substantially increased TEE-risk (OR 8, *p* = 0.042) in the PICC-group versus in the PORT-group, prior to RC. Hence, 38 patients with PICC suffered a minimum of one TEE, compared to one patient with PORT, during NAC. In addition, more patients in the control group, with neither PICC nor PORT, developed a TEE (*n* = 3), than those registered in the entire PORT-group in the defined timeframe. In the 38 PICC-patients with TEE, PEs were most common (47%). However, a total of 13 patients (29%) developed TEE anatomically related to the CVA, of which four patients had both PE and CVA-thrombosis. Both TEE-types mentioned required immediate intervention (CTCAE 3). Overall, 71% (*n* = 32) of the TEEs in the PICC-group were of a higher severity (CTCAE 3–4). No significantly higher TEE-risk was seen post-RC. This result was expected, due to PICC or PORT being customarily removed perioperatively, and could thus be interpreted as an additional argument concerning the impact and importance of the location of CVA-placement during NAC. As the initial choice of CVA is much dependent on the center’s routines, we suggest that our findings could lead to further discussions for stricter guidelines, whether PORTs should become the standard and first choice for NAC-administration in MIBC-patients or not.

Total TEE-incidence in the entire patient cohort was 18% (*n* = 67), with a total number of 83 TEEs, including some patients suffering multiple events. A total number of 57 TEEs were registered within the NAC-group, with 75% (*n* = 43) occurring pre-RC. This incidence-rate is lower than in some studies, e.g., works of Ottosson et al. or Dyer et al. (35% and 24%, respectively). However, their observation time exceeded ours, which we limited to 30 days post-RC in order to investigate TEE in near time proximity to NAC-administration [23,24]. The investigations of Duivenvoorden et al., with a timeframe spanning six months post-RC, showed a more similar result (14%), with 58% occurring pre-RC [25]. Zareba et al. shared timespan with our study and reported a total TEE-incidence of 19% in the NAC-administered group, of which 75% occurred pre-RC. Both studies had a more extensive patient exclusion, not incorporating those already on anticoagulation therapy and Zareba et al. did not include patients with a previous history of VTE, only registering TEEs if radiologically verified, perhaps leading to a lower TEE-incidence than our presented findings [12]. Ottosson et al. showed a significantly increased risk for TEEs amongst NAC-administered during the period from TUR-B to cystectomy (OR 8) and near in time to NAC-administration. The authors suggested that CVA-type might mainly influence TEE-incidence as they detected a discrepancy between TEEs amongst PICC-and PORT-patients [23]. When introduced into a vein, a CVA will both reduce the blood velocity and generate an endothelial damage. As PICCs are inserted into v. brachialis, a smaller vein compared to PORTs v. subclavia, the CVAs unwanted secondary effects might become more prominent, perhaps leading to the amplified risk for vascular thrombosis [26].

To fully receive the benefits of NAC, all pre-planned cycles should be provided. If a patient is deemed unfit to continue with chemotherapy by the multidisciplinary conference after 1–2 cycles, NAC is discontinued, and instead directly scheduled for RC. Thus, leaving patients with suboptimal NAC. Discontinued NAC, with only 1–2 cycles, could thus be considered to having been provided in vain. A total of 10 patients in the cohort had their NAC terminated, as well as suffered a TEE pre-cystectomy. As many as 60% of these patients had to end their chemotherapy due to their TEE, whilst the remaining TEE-patients ended NAC because of other, non-TEE-related, circumstances. In addition, every NAC-terminated patient who suffered a PE (*n* = 3) had their TEE as sole reason for termination, whereas other MDT-decisions were based upon the occurrence of TEE as well as general impaired condition. The results are worrisome, as PEs were the cohort´s most frequent TEE-type. Details of NAC-terminations are presented in Table 4.

PORTs are generally more tolerated by their carriers, with PORT-patients perceiving their CVA as less of an interference with day-to-day life. PORTs are also preferred by staff, regarding it as a safer choice for patients. Reasons for the change to more frequent use of PICCs could be the general perception of PICCs as being more timesaving, with the advantages of not requiring scheduled OR-time during implantation, combined with the widespread notion of PICCs being more cost-efficient. Cost analysis by two previous RCTs have challenged the perceived cost-benefit concept, and could instead present that PICCs were more expensive, if adjusted for CVA-days. Both RCTs contributed higher costs for PICCs to TEE-related complications. The study populations of the RCTs were considerably diverse, including patients that had received either adjuvant chemotherapy or palliative care and were treated for multiple types of malignancies [26,27,28]. We suggest hypothetically that the cost-efficiency of PORTs could potentially become even greater if applied to our cohort due to the established high incidence of TEE-complications in MIBC-patients.

PORTs are generally associated with few AEs, yet Taxbro et al. exhibited that local infections are more common in PORT- than in PICC-patients. However, the overall infection-frequency is low enough amongst both CVA-types that the risk of infection alone cannot motivate disfavoring PORTs over PICCs. Infections amongst PORT-patients have been speculated to be a consequence of staff lacking clinical knowledge, resulting in suboptimal hygiene standards [29]. The same argument could be applied to PICCs, reasoning that if staff routines for PICC-placement were improved, the AE-incidence might be decreased. However, in our cohort, in which the investigated AE was TEE, the incidence was constant during the observed years. Thus, presenting that although clinical procedures may improve over the years, no difference in the number of TEEs could be noted in our material. TEE-incidences over the observation time of the cohort are presented in Figure 4.

Furthermore, a secondary objective in our study was an analysis of thrombosis prophylaxis and its role in preventing TEEs. No significant relationships between preoperative TEE-risk and antiplatelet or anticoagulant drugs were found. Opinions differ regarding thrombosis prophylaxis during malignancies, with some studies showing a TEE-preventative effect of antiplatelet over anticoagulant, and others showing no such relationship, this in accordance with our findings [12,30]. In addition, findings by Eriksson et al. presented that acute kidney injury and chronic kidney-disease were seen in 41% and 11%, respectively [11]. Mehrazin et al. could in a recent study show that thrombosis prophylaxis which are administered post-RC IN MIBC-patients, often becomes subtherapeutic. NAC-treatment, as well as the bladder malignancy, can both independently contribute to decreased kidney function, reducing the effect of low molecular weight heparin (LMWH), as the prophylaxis requires an adequate renal clearance. Altogether, the argument to put MIBC-patients on thrombosis-prophylaxis as the main preventative measure against TEEs seems to be invalid. LMWH can instead become counterproductive, as it results in a heightened risk for bleeding [31].

A weakness in our study is the retrospective nature, yet a recent study indicates that retrospective studies can provide as good results as their prospective counterparts, if based on a pre-set variable list, including fixed variables that would be the same regardless of prospective or retrospective collection [32]. Another weakness could be missing data on some patients due to unavailable medical records if they, e.g., had received NAC at a smaller local hospital, leading to additional TEE perhaps being unregistered. Some data regarding the TEE-risk post-cystectomy were not collected, such as operation time or infectious complications, but could potentially contribute to the incidence of TEE and may be investigated further in future analyzes. In addition, the PORT-group included in the analysis is relatively small with few events. However, it remains representative of the included cystectomy centres patients, as all patients observed during the cohort’s time frame have been incorporated. If additional centres could have participated and more patients could have been added to the PORT-group, perhaps our findings could be even more evident. Also, if a greater cohort could be assembled, even more variables could have been used in the adjusted log rank tests. The participating centers were all located in Sweden, and therefore the results may not necessarily be directly transferable to patients in other countries. However, as many other studies have their patient-data retrieved from national databases, our results could be considered more comprehensive as we have analyzed data from a clinical database, based on individual and meticulously validated medical records of each patient.

## Figures and Tables

**Table 1:** Baseline characteristics on the study cohort.

		CVA-Group	CVA-Group	CVA-Group
		PICC *n* = 235	Port *n* = 42	Neither *n* = 90
Variable		Mean (SD)	Mean (SD)	Mean (SD)
Age		67 (7)	69 (7)	66 (7)
BMI		26 (4)	26 (3)	26 (4)
CACI		5 (1)	5 (1)	4 (1)
no. NAC-cycles		3 (1)	3 (1)	0 (0)
		*n* (%)	*n* (%)	*n* (%)
**Sex**				
	Female	57 (24)	9 (21)	18 (20)
	Male	178 (76)	33 (78)	72 (80)
**Center**				
	Umeå	135 (57)	5 (12)	22 (24)
	Sundsvall	39 (17)	1 (1)	9 (4)
	Västerås	5 (2)	32 (76)	10 (11)
	Linköping	56 (24)	4 (10)	49 (54)
**ASA**				
	1	34 (12)	3 (7)	22 (24)
	2	140 (60)	28 (67)	54 (60)
	3	61 (26)	11 (26)	14 (16)
**Current Smoker**		24 (10)	3 (7)	8 (9)
**cT-Stage**				
	T2	147 (63)	23 (55)	59 (66)
	T3	73 (31)	16 (38)	30 (33)
	T4a	15 (6)	3 (7)	1 (1)
**Thrombosis Prophylaxis**				
	Anticoagulant	16 (7)	4 (10)	4 (5)
	Antiplatelet	40 (17)	5 (12)	14 (16)

Percentage (%) calculated from total amount (*n*) in each CVA-group. CVA; central venous access. PICC; peripherally inserted central catheters. PORT; centrally inserted totally implanted vascular access port-a-cath. BMI; Body mass index. ASA; American Society of Anesthesiologists Physical Status. CACI; Carlson Comorbidity Index. cT-stage; clinical tumor-stage. No. NAC-cycles; number of neoadjuvant chemotherapy-cycles.

**Table 2:** Characteristics of TEEs from TUR-B–cystectomy.

Variable		CVA Group PICC *n* (%) *n* = 235	CVA Group Port *n* (%) *n* = 42	CVA Group Neither *n* (%) *n* = 90
**Patients with TEE**		38 (16)	1 (2)	3 (3)
**Type of TEE**				
	DVT	3 (7)	0 (0)	2 (50)
	Thrombophlebitis	7 (16)	0 (0)	0 (0)
	PE	21 (47)	1 (50)	2 (50)
	From CVA	13 (29)	1 (50)	0 (0)
	Stroke/TIA	0 (0)	0 (0)	0 (0)
	Angina/MI	1 (2)	0 (0)	0 (0)
	Total amount	45 (100)	2 (100)	4 (100)
**CTCAE**	1	0 (0)	0 (0)	0 (0)
	2	13 (29)	0 (0)	0 (0)
	3	29 (64)	2 (100)	3 (75)
	4	3 (7)	0 (0)	1 (25)
	5 Total amount	0 (0) 45 (100)	0 (0) 2 (100)	0 (0) 4 (100)

TEE; thromboembolic event. DVT; deep vein thrombosis. PE; pulmonary embolism. CVA; central venous access. TIA; transitory ischemic attack. MI; myocardial infarction. CTCAE; common Terminology Criteria for Adverse Events.

**Table 3:** *p*-values of analyzed TEE-risk between CVA-groups.

Time Period	Compared CVA-Groups	*p*-Value
TURB-Cystectomy		
	PICC-PORT	0.042
	PICC-Neither	0.03
	PORT-Neither	0.745
Cystectomy-30 days post-cystectomy		
	PICC-PORT	0.925
	PICC-Neither	0.995
	PORT-Neither	0.955
TURB-30 days post-cystectomy		
	PICC-PORT	0.06
	PICC-Neither	0.028
	PORT-Neither	0.657

PICC; peripherally inserted central catheters. PORT; centrally inserted totally implanted vascular access port-a-cath. TEE; thromboembolic event. CVA; central venous access. TURB; transurethral resection of bladder.

**Table 4:** Patients with a preoperative TEE and terminated NAC.

				No. Planned		
Patient	CVA	NAC-REGIMEN	No. Cycles	Cycles	Type of TEE	Reason for Termination
1	PICC	MVAC	3	4	PE	TEE
2	PORT	MVAC	2	3	PE, from CVA	TEE
3	PICC	MVEC	2	3	from CVA	TEE + impaired general condition
4	PICC	MVAC	1	3	from CVA	TEE + impaired general condition
5	PICC	OTHER	2	*	from CVA thrombophlebitis	septic infection
6	PICC	MVAC	2	3	PE, from CVA	TEE
7	PICC	CARBO-GEM	2	3	DVT, from CVA	TEE + impaired general condition
8	PICC	MVAC	1	4	thrombophlebitis	impaired general condition
9	PICC	CARBO-GEM & OTHER	2	*	from CVA	neutropenia
10	PICC	OTHER	3	4	thrombophlebitis	reduced kidney function

Patients with terminated NAC due to a preoperative TEE are stated in bold. *; not specified. PICC; peripherally inserted central catheters. PORT; centrally inserted totally implanted vascular access port-a-cath. No. NAC-cycles; number of neoadjuvant chemotherapy-cycles. MVAC; Methotrexate-Vinblastine-Doxorubicin-Cisplatin. MVEC; Methotrexate-Vinblastine-Epirubicin-Cisplatin. CARBO-GEM; Carboplatin-Gemcitabine. OTHER; other types of regimens, such as Gemcitabine-Cisplatin. TEE; thromboembolic event. DVT; deep vein thrombosis. PE; pulmonary embolism. From CVA; TEE anatomically related to central venous access.
